# Deciphering the roles of myeloid derived suppressor cells in viral oncogenesis

**DOI:** 10.3389/fimmu.2023.1161848

**Published:** 2023-03-23

**Authors:** Alexander Glover, Zhaoqin Zhang, Claire Shannon-Lowe

**Affiliations:** Institute of Immunology and Immunotherapy, The University of Birmingham, Birmingham, United Kingdom

**Keywords:** myeloid derived suppressor cells (MDSC), epstein barr virus (EBV), human papillomavirus - HPV, viral hepatitis, viral oncogenesis, immunotherapy, neutrophils, monocytes

## Abstract

Myeloid derived suppressor cells (MDSCs) are a heterogenous population of myeloid cells derived from monocyte and granulocyte precursors. They are pathologically expanded in conditions of ongoing inflammation where they function to suppress both innate and adaptive immunity. They are subdivided into three distinct subsets: monocytic (M-) MDSC, polymorphonuclear (or neutrophilic) (PMN-) MDSC and early-stage (e-) MDSC that may exhibit differential function in different pathological scenarios. However, in cancer they are associated with inhibition of the anti-tumour immune response and are universally associated with a poor prognosis. Seven human viruses classified as Group I carcinogenic agents are jointly responsible for nearly one fifth of all human cancers. These viruses represent a large diversity of species, including DNA, RNA and retroviridae. They include the human gammaherpesviruses (Epstein Barr virus (EBV) and Kaposi’s Sarcoma-Associated Herpesvirus (KSHV), members of the high-risk human papillomaviruses (HPVs), hepatitis B and C (HBV, HCV), Human T cell leukaemia virus (HTLV-1) and Merkel cell polyomavirus (MCPyV). Each of these viruses encode an array of different oncogenes that perturb numerous cellular pathways that ultimately, over time, lead to cancer. A prerequisite for oncogenesis is therefore establishment of chronic infection whereby the virus persists in the host cells without being eradicated by the antiviral immune response. Although some of the viruses can directly modulate the immune response to enable persistence, a growing body of evidence suggests the immune microenvironment is modulated by expansions of MDSCs, driven by viral persistence and oncogenesis. It is likely these MDSCs play a role in loss of immune recognition and function and it is therefore essential to understand their phenotype and function, particularly given the increasing importance of immunotherapy in the modern arsenal of anti-cancer therapies. This review will discuss the role of MDSCs in viral oncogenesis. In particular we will focus upon the mechanisms thought to drive the MDSC expansions, the subsets expanded and their impact upon the immune microenvironment. Importantly we will explore how MDSCs may modulate current immunotherapies and their impact upon the success of future immune-based therapies.

## Introduction

The concept that an infectious agent may be causal in oncogeneses dates back to 1842, when Domenico Rigoni-Stern described cervical cancer incidence as being highest in sex workers, lowest in nuns and at an intermediate level in married women (1). However, it took until 1911 for the first tumour-causing virus to be identified; discovered by the American pathologist Peyton Rous, the Rous sarcoma virus was shown to be responsible for the carcinogenesis of domestic chicken tumours (2). The first human cancer causing virus, the Epstein-Barr virus (EBV, was identified in 1964 as the infectious agent responsible for endemic Burkitt lymphoma (3). The later surprise discovery that EBV infects more than 95% of the world’s population and is the causal agent of infectious mononucleosis (IM) illustrates the concept that a common virus can trigger rare cancers (4). There are now seven known human oncogenic viruses (5, 6). They include the human gamma herpesviruses; EBV and Kaposi’s Sarcoma-Associated Herpesvirus (KSHV), members of the high-risk human papillomaviruses (HPVs), hepatitis B and C (HBV, HCV), Human T cell lymphotropic virus (HTLV-1) and Merkel cell polyomavirus (MCPyV). In no case does triggering cancer form part of the virus’ natural life cycle, yet all the viruses share the ability to cause chronic infection, which over time and with additional risk factors can lead to carcinogenesis (5).

Although it is well documented that virus-associated cancers are common in those with immunocompromise (7), particularly in conditions such as Kaposi’s sarcoma or post-transplant lymphoproliferative disease (PTLD), viral oncogenesis also occurs in those with an intact immune system. The mechanisms enabling viral oncogenesis are complex and varied but often involve modulation of the antiviral immune response, including limited expression of immunogenic viral antigens, or infection of tissues with reduced immune surveillance. There is, however, growing evidence that myeloid-derived suppressor cells (MDSCs) may play a role in this process.

MDSCs are a heterogeneous group of myeloid cells capable of suppressing antigen-specific immune function. There are three major classes of MDSCs the phenotype of which is illustrated in **Table 1**. Polymorphonuclear (PMN-MDSCs) resemble neutrophils but have a suppressive function. While key differences in function have been described between PMN-MDSC and neutrophils in both mice and humans (8), other groups have called into question if they can be truly be described as a differentiated population rather than an alternative activation state (9). Monocytic (M-MDSCs) are morphologically identical to monocytes but are immunosuppressive and HLA-DR^low^. Early MDSCs (e-MDSCs) are the third group of suppressive myeloid cells; lacking mature monocyte or neutrophil markers, it is unclear if these cells could be the precursors for other MDSC classes (10). The study of MDSCs has been hampered by a lack of a specific phenotype to separate them from conventional neutrophils and monocytes (11). Accurate identification requires either demonstration of suppressive function, use of biochemical surrogate markers or density centrifugation in the case of PMN-MDSCs, making identifying populations in fixed tissues difficult (11). In addition, technical differences between laboratories can introduce variation in MDSC enumeration (12).

**Table 1:** Phenotype and functional markers by MDSC class.

Cell type	Human surface phenotype	Functional Characteristics	Functional Markers
PMN-MDSC	CD11b^+^ CD14^-^CD15^+^ CD66b^+^ Low Density^1^	T Cell suppression	ARG-1, NO, ROS, STAT3,
e-MDSC	Lin^-^ CD33^+^ HLA-DR^-^ CD14^-^ CD15^-^		
M-MDSC	CD14^+^ CD15^-^ HLA-DR^low/-^	T cell suppression Differentiation to TAMs	ARG-1, NO, ROS, STAT3, IL-10, TGF-ß, PD-L1

1- Note phenotype for PMN-MDSC is identical to mature neutrophils and therefore requires further isolation based on density.

MDSCs were first described in the context of cancer under a variety of names before the current terminology was settled upon (13). In cancer, MDSCs are associated with a reduction in overall (OS) and progression-free survival (PFS) and an increased rate of relapse (14). Over time they have been identified in a greater variety of disease states including infection (15), pregnancy (16), obesity (17) and sepsis (18). We will first discuss the mechanisms behind MDSC generation and how this can prevent viral clearance by the adaptive immune system. We will then discuss the evidence for MDSCs for each of the cancer-causing viruses. Finally, we will discuss what therapeutic options are available to eliminate MDSC populations.

## Generation and function of MDSCs

### PMN-MDSC

While functionally distinct, PMN-MDSC are phenotypically almost identical to conventional neutrophils and morphologically resemble either immature band cells or have the segmented nucleus of a mature neutrophil. Neutrophils are the most abundant leukocyte in the blood and under steady-state conditions are produced in the marrow from lineage-committed precursors (19). The precise mechanisms by which PMN-MDSC are generated rather than inflammatory activated neutrophils are not fully understood.

Neutrophils are typically seen as short-lived cells, but their lifespan can be extended under inflammatory conditions. In particular, PGE2 can suppress neutrophil apoptosis and prolong survival through activation of protein kinase A (PKA) (20). Neutrophils have a large variety of cell surface receptors for chemokines, cytokines and immunoglobulin as well as innate immune receptors which act through diverse signalling pathways to trigger homing and activation (21). Classically, neutrophils extravasate at sites of inflammation following a chemokine gradient and are then activated after exposure to damage-associated molecular patterns (DAMPs) and pathogen-associated molecular patterns (PAMPs). These activated, or effector, neutrophils then kill pathogens by phagocytosis, NETosis and degranulation (19, 22) and therefore constitute the immunological first line of defence against bacterial and fungal infections.

The process of MDSC generation appears to involve two distinct steps: the production of myeloid progenitors and then activation to a full PMN-MDSC phenotype. The relative contribution of each step may then impact on the morphological maturity of the resulting cells. Granulocyte differentiation is initially triggered by G-CSF which can result in emergency myelopoiesis and the release of immature myeloid cells (23). Thereafter, a number of factors have been suggested to trigger the generation of MDSCs from progenitors, including Stem cell factor (SCF) acting *via* C-KIT (24), combinations of G-CSF/IL-6 acting through C/EBPβ (25) and S100A8/A9 through NFκB. Furthermore, factors produced by MDSCs such as S100 family proteins can trigger an autocrine feedback loop leading to further MDSC generation (26). However, other studies propose mature blood neutrophils can develop an immunosuppressive phenotype induced by factors such as PGE2 (27) or endoplasmic reticulum (ER) stress (28) triggered by hypoxia, nutrient deprivation or increased ROS and NOS often associated with chronic inflammation and in a tumour microenvironment (29, 30).

PMN-MDSCs are usually characterised by their low density as opposed to the high density of classical mature neutrophils, although some activated pro-inflammatory neutrophils are also low density. Gene expression profiling of low-density neutrophils identified the expression of LOX-1 in the blood of cancer patients as well as in tumour tissue but not in healthy donors (28). Initially, due to their band-like immature morphology, similar to that observed in LOX1+ neutrophils, it was proposed that PMN-MDSCs have an immature phenotype (28). Yet, patients treated with high-dose GCSF for stem cell mobilisation exhibit an expansion of activated CD10+ neutrophils that are morphologically mature; inhibit T cell proliferation and release IFN-γ *in-vitro*, and have an activated phenotype (31). While this appears to be a contradiction, a further study correlated CD11b and CD16 expression with nuclear morphology and maturity demonstrating both mature and immature cells can be suppressive and meet the criteria to be defined as PMN-MDSCs. However, the greatest degree of immune suppression was seen in CD11b^+^CD16^+^ mature cells (32).

### M-MDSC

Monocytes differentiate from myeloid precursors in the marrow under the control of growth factors and are then released into the circulation. Conventional monocytes are divided into classical (CD14+CD16-), non-classical (CD14dimCD16+) and intermediate (CD14+CD16+) populations. Classical monocytes are produced from the marrow and are capable of phagocytosis and tissue migration. As they mature, they develop an intermediate phenotype which is strongly HLA-DR+ and can act as antigen-presenting cells. Non-classical monocytes principally function through complement and antibody-dependent phagocytosis (33). Monocytes can then enter tissues and further differentiate into macrophages and dendritic cells (34). However, M-MDSC arise from the CD14+ population and are HLA-DR negative (35). They express increased IL-10 and IL-8 but reduced IL-1β, IL-6 and TNF compared to classical monocytes (35).

Monocyte activation towards an inflammatory phenotype occurs after exposure to PAMPs and DAMPS. Conversely, M-MDSC generation appears to occur after weaker activation signals by cytokines, particularly TGF-β, or repeated low-intensity toll-like receptor (TLR) stimulation. TGF-β initially appears to act as a chemoattractant for CD14+CD33+ myeloid cells. Tumour tissue from multiple different cancers express TGF-β and this expression is closely associated with infiltration of CD14+CD33+ myeloid cells. In addition, TGF-β also appears to play a role in acquisition of the M-MDSC phenotype as demonstrated by *in-vitro* treatment of CD14+ monocytes with TGF-β, GM-CSF and IL-6, resulting in the development of the suppressive phenotype. Furthermore, repeated TLR 2/4 stimulation of the monocytes triggers downregulation of HLA-DR and secretion of IL-10 and TGF-β, thereby amplifying the M-MDSC phenotype and generating a TGF-β-mediated feedback loop (36, 37). In monocytes, TLR stimulation has been demonstrated by tumour-derived extracellular vesicles containing factors such as HSP72 that drive M-MDSC generation *via* MyD88/STAT3 requiring autocrine IL-6 production (38). In addition, WNT5a, S100A8/A9, Il-4 and PGE2 have variously been linked to the acquisition of an MDSC phenotype (39–42). However, maintenance and accumulation of the M-MDSC population appears to be mediated, at least in part, by PD-1 and TNF signalling respectively, whereby PD1-deletion in a murine model appears to prevent M-MDSC accumulation (43) and TNF signalling through TNFR-2 appears to prevent M-MDSC apoptosis in a c-FLIP-dependent manner.

M-MDSCs can further differentiate into tumour-associated macrophages (TAMs) which have an immunosuppressive M2 phenotype (44) that secrete IL-10, express PD-1 and contribute to tumour angiogenesis, and appears to depend on S100A9 expression and signalling acting *via* C/EBPbeta (39).

### E-MDSC

E-MDSC have been identified as myeloid cells lacking monocyte or neutrophil markers both in the peripheral blood and the tumour microenvironment. Due to the lack of mature lineage markers, they are thought to be immature and are potentially precursors for the other MDSC subsets. *In-vitro* e-MDSC show the least capacity to suppress T cell proliferation or IFN-γ release (32). No correlation between e-MDSC frequency and OS has been shown for either a head and neck or ovarian cancer (32, 45). One concern with e-MDSC enumeration is that mature basophils have a similar phenotype but are not immunosuppressive. Using a basophil marker, such as CD123^high^ expression, allows more accurate identification of e-MDSC (10). More accurate identification of e-MDSC may help future studies to explore their functional effects.

### Mechanisms of immunosuppression

The method by which MDSCs trigger immunosuppression depends on the class of cell involved, but common to both PMN-MDSCs and M-MDSCs is the secretion of soluble factors. This effect appears to be the primary method by which PMN-MDSCs mediate immunosuppression and is stronger on a per-cell basis compared to M-MDSCs. Arginase is located in the azurophilic granules of neutrophils and is secreted by both activated neutrophils and MDSCs (46). Arginine is essential for T cell proliferation and function (47). When PMN-MDSCs are cultured with T cells inhibition of arginase restores proliferation, enhances IFN-γ production and improves cytotoxic function (48) (32). Inhibiting the function of iNOS can similarly reverse the effect of PMN-MDSC and M-MDSCs on T cell proliferation (32, 49). ROS is produced by both M-MDSCs and PMN-MDSCs and can inhibit antigen-dependent T cell proliferation (50, 51). ROS is produced under the control of STAT3 and appears to function in the immunological synapse as the expression of the integrin MAC-1 is required for its effect on T cells (50).

M-MDSCs have several additional functional roles. M-MDSCs can induce TRegs through a CD40-CD40L dependent process (52) and trigger TReg and PMN-MDSC recruitment by secreting CCL2, CCL4 and CCL5 (53, 54). Secretion of cytokines including IL-10 and TGF-β promotes tolerance, suppresses cytotoxic function and contributes to further MDSC generation (42, 55). M-MDSCs also express PD-L1 and can adversely affect immune checkpoint therapy (56, 57)

MDSCs may have a physiological role to control inflammation, particularly in the lung and liver. Lung resident neutrophils have a reduced cytokine response to LPS challenge and can protect the lung from harmful inflammation (27). Furthermore, in a model of asthma, PMN-MDSCs can inhibit the function of Th2 cells and suppress inflammation (58) in a PGE2 and COX-1 dependent manner. However, in COVID-19 early expansion of MDSCs is predictive of a poor outcome by preventing viral clearance, yet in late disease, they can be beneficial by reducing secondary inflammation (59). As will be discussed later MDSCs can be beneficial in preventing inflammation in chronic viral hepatitis (60). Although, in the longer term this may be deleterious as it would inhibit viral clearance and promote carcinogenesis. Both examples illustrate the yin and yang of MDSC function depending upon the state of an infection.

### Conclusion on MDSC generation and function

**Figure 1:**
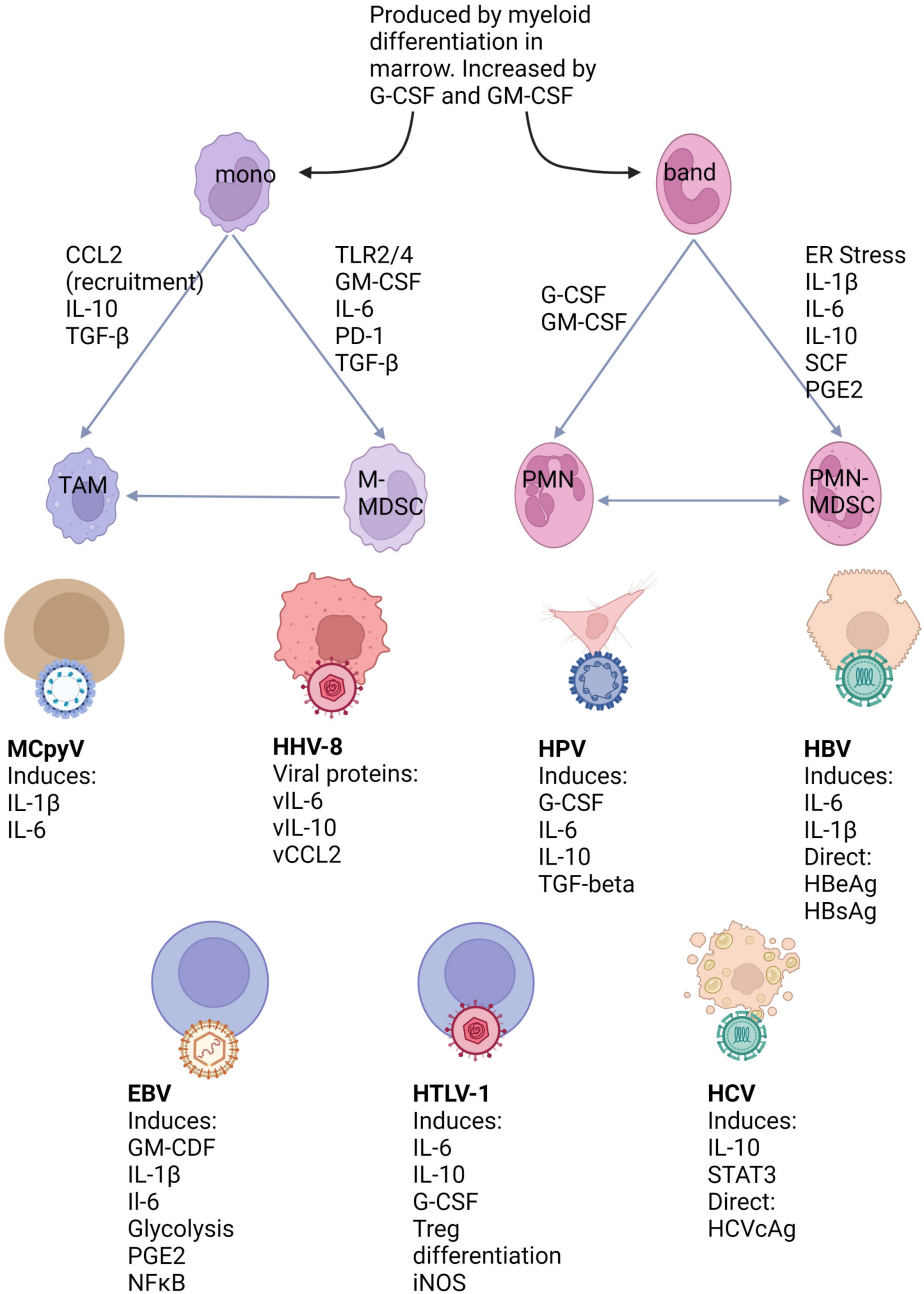
Mechanisms of MDSC generation and their relation to virus associated cancers. Myeloid cells are produced during myelopoiesis in the marrow in both steady state and inflammatory conditions. However, myeloid differentiation can be greatly expanded under growth factor control and lead to the release of immature cells into the circulation. Under conditions favouring MDSC development both bands and mature neutrophils can be induced to this phenotype. M-MDSCs arise from classical monocytes and can also differentiate to TAMs. The human oncogenic viruses illustrated below can either directly or indirectly produce factors known to be linked to MDSC differentiation pathways.

MDSCs are generated in response to chronic inflammation, a state which is seen in all virally induced cancers. The action of cytokines, particularly IL-6 and TGF-β, as well as growth factors such as GM-CSF are important in both triggering emergency myelopoiesis and promoting the acquisition of an MDSC phenotype. Factors such as ER stress appear important in PMN-MDSC generation, whereas TLR stimulation and TGF-β are more important for M-MDSCs. MDSCs can also act *via* autocrine feedback loops to amplify signals leading to their generation and recruitment. MDSCs can then act to suppress T cell function through a variety of interconnected mechanisms. The nature of the underlying immune microenvironment can influence if M-MDSCs or PMN-MDSCs are predominant. **Figure 1** links factors produced by oncogenic viruses or infected/transformed cells with the pathways which result in MDSC generation.

As discussed above identifying MDSCs can be difficult. PMN-MDSCs are short-lived labile cells that cannot be frozen so cannot be studied retrospectively (12). However, progress is being made to establish common flow cytometry protocols (12) and multiplex tissue staining panels (61) to ensure the unambiguous identification of the different MDSC populations, neutrophils and monocytes. This is particularly important when understanding the role of the different MDSC subsets in the context of different cancers. Although MDSCs have not yet been considered in many of the virus-associated cancers, the tumour microenvironment of these cancers actually exhibit the distinct characteristics that are both conducive to the generation of MDSCs and consistent with their presence, and therefore warrant further investigation.

## Oncogenic viruses

### Human T cell lymphotropic virus-1 (HTLV-1)

Human T-lymphotropic virus-1 (HTLV-1) is a complex retrovirus which is prevalent worldwide in clusters of high-incidence areas, such as Japan and the Caribbean (62). Initial infection occurs either in infancy, due to spread in breastmilk, or as a sexually transmitted infection. Following a prolonged asymptomatic latency period, HTLV-1 can cause adult T cell leukaemia/lymphoma (ATL), occurring in 2-5% of carriers, as well as inflammatory diseases such as HTLV-1-associated myopathy (HAM).

HTLV-1 transmission is primarily dependent upon direct cell-to-cell contact *via* a virological synapse rather than infection of target cells with cell free virus. Following infection HTLV-1 integrates into the target cell DNA and is thought to persist by infectious (*de novo*) spread during early infection and thereafter by mitotic expansion (infected cell proliferation) of cells containing integrated HTLV-1 genome. Over time, the proviral load rises commensurate with the number of HTLV-1 infected clones and the subsequent risk of oncogenic transformation (63). HTLV-1 modifies the infected cell through the action of two genes, TAX and HBZ, that drive proliferation, inhibit apoptosis and promote tissue migration and are both required for oncogenesis (64). TAX is a multifunctional protein capable of driving profound changes to the immunological microenvironment.

ATL is a highly aggressive malignancy associated with profound immunosuppression, largely mediated through the action of TAX. Indeed TAX drives the acquisition of a CD4+CD25+ Treg phenotype of the infected transformed cells (65) and triggers the upregulation of MHC class II allowing infected cells to acquire a tolerogenic antigen-presenting cell function (66). TAX also drives changes to the cytokines secreted by the infected cells, leading to the production of inflammatory cytokines such as TNF-α and IL-6 as well as anti-inflammatory IL-10 through HBZ induction of TIGIT (67, 68). Much of the work looking at the tumour microenvironment has focused on lymphocytes where HTLV-1+ non-tumour cells assist in the growth of tumour cells particularly through IL-10 production (69). Checkpoint blockade, particularly the PD-1 axis leads to disease flair, presumably related to the Treg nature of the tumour (70). Allograft is the only curative treatment, implying that a graft versus tumour effect is important (71). Immunotherapy directly targeting viral antigens is at a much earlier stage, but animal studies show in principle this could be effective (72). Antiviral therapy with zidovudine and interferon is effective in ATL, particularly in non-lymphomatous disease (73) and although the mechanism behind this is not fully understand, it is likely mediated by reducing *de novo* infection of bystander cells and therefore modulating the TME.

There is very little data on MDSCs in HTLV-1 infection. Neutrophilia is common in ATL, particularly in the leukaemic form (74), and indeed neutrophils appear to show spontaneous activation in HTLV-1 infection with the production of IFN-γ, TNF-α and IL-12 and increased oxidative stress (75–77). Mechanistically, TAX is likely to drive both neutrophilia and neutrophil activation; TAX expressing cells produce G-CSF, leading to neutrophilia and splenomegaly in xenograft models (78) and TAX can also induce the expression of iNOS through an NFκB dependent pathway (79). M2 macrophages in the TME are associated with a worse prognosis in ATL and promote the growth of malignant cells *in-vitro* (80). However, there is almost no work focusing on MDSC in HTLV-1 infection or attempting to characterise the expanded myeloid compartment. Given the combination of chronic inflammation, production of regulatory cytokines and neutrophil expansion, the presence of MDSCs would certainly be very plausible. How these would function alongside a tumour composed of regulatory T cells is unclear but understanding this may help design more effective immunotherapy.

### Merkel cell polyomavirus (MCPyV)

Merkel cell polyomavirus (MCPyV) is one of 14 human polyomaviruses, but surprisingly is the only one to drive carcinogenesis (81). MCPyV is responsible for up to 80% of cases of Merkel cell carcinoma (MCC), a rare and highly aggressive skin malignancy seen in older age. Initial infection with MCPyV appears to be completely asymptomatic and occurs during childhood, with a seroprevalence of around 70% (82). However, oncogenesis is always associated with viral integration into the host cell DNA, UV exposure and immune senescence. The MCPyV genome is always mutated in MCC resulting in truncation of the Large T (LT) gene that preserves the Retinoblastoma (RB) binding domain but removes its virus replication functions, resulting in a replication-deficient virus. Preservation of the LT RB binding domain and expression of Small T (ST) ensures continual inactivation of RB and degradation of P53 *via* ubiquitination by MDM2 respectively, leading to uncontrolled cell cycle, accumulation of mutations and development of MCC. Interestingly, both RB and P53 are highly mutated in MCPyV-negative MCC that more closely resemble the UV mutational signature observed in malignant melanoma (81) highlighting how the viral oncogenes mimic the mutational signature in the virus-negative carcinoma counterparts.

Immune control of MCPyV appears to be important in preventing carcinogenesis. Individuals with immunosuppression driven by factors such as HIV, ageing or medication exhibit significant increases in the frequency of MCC (81). However, MCPyV is largely controlled in healthy infected individuals who have readily detectable CD4+ T cells with specificity against LT and ST antigens (83). Conversely, although a clonal CD3+, CD8+ immune infiltrate into the tumour microenvironment is associated with a better prognosis (84), the T cells show signs of exhaustion with the expression of PD-1 on infiltrating lymphocytes and PD-L1 in the tumour microenvironment (85). However, reversing T cell anergy by blocking the PD-1 axis with pembrolizumab has revolutionised the treatment of MCC (86). In MCPyV+ MCC the response rate is around 70%, with 30% achieving CR and responders showing 89.6% survival at 3 years (87). Interestingly, while there was no correlation between CD8+ infiltrate, PD-L1 expression and response to PD-1 blockade a higher neutrophil-to-lymphocyte ratio in the blood was associated with a lower response rate and poorer overall survival (86). Unfortunately, neutrophil phenotyping was not performed in this study to assess if these cells were in fact PMN-MDSCs.

There are no studies, which have studied MCC with histological panels designed to enumerate MDSCs. Similarly in the blood, a high neutrophil to CD8+ ratio in the tumour is a poor prognostic marker (88). PD-L1 is expressed on tumour infiltrating cells expressing CD11c+, CD162+ and CD33+, attributed to DCs and macrophages but some CD33+ cells may be MDSCs (85, 89). PD-L1+CD33 positive cells appear to be enriched in the periphery of MCC tumours and may function to shield the tumour against infiltrating PD-1+ lymphocytes (89). In addition, MCC strongly express CD200R, which is associated with tumour-infiltrating myeloid cells including M2 macrophages and MDSCs (90, 91).

Overall, the understanding of non-lymphoid cells in the microenvironment of MCC is at a limited level, with evidence that myeloid cells in the form of circulating neutrophils and infiltrating PD-L1 cells may inhibit T cell response to tumour antigens. No current biomarkers accurately predict response to checkpoint inhibition therapy (92). Understanding the TME is likely to be of significance as there is currently work to target MCPyV antigens with therapeutic vaccination or adoptive immunotherapy, which has shown some success in small early-phase studies (93) (94).

### Kaposi’s sarcoma-associated herpesvirus (KSHV/HHV-8)

Human herpesvirus-8, also known as the Kaposi sarcoma associated herpesvirus, is linked to two distinct human malignancies- Kaposi sarcoma (KS) and primary effusion lymphoma (PEL) (95). In endemic areas, HHV-8 infection usually occurs in childhood through infected saliva, but sexual transmission is an important route of spread in other populations. HHV-8 is endemic only in limited areas, particularly sub-Saharan Africa, the Mediterranean and south America (96). HHV-8 primarily infects B cells and epithelial cells before establishing latency in B lymphocytes and monocytes (97). Subsequent oncogenesis appears dependent on viral reactivation.

HHV-8 is a large double-stranded DNA virus that dedicates at least 20% of its genome to modulating the cell cycle and antiviral immunity and encoding numerous viral homologues of human genes. Latent cycle genes such as LANA-1, v-cyclin and v-FLIP as well as lytic cycles genes such as v-IL-6 and v-bcl-2 appear important in carcinogenesis, although the relative dependency on individual genes differs depending on the malignancy (97).

KS was historically a rare tumour but incidence rose rapidly in the 1980s with the emergence of HIV/AIDS, indicating the importance of immune control of the virus in preventing oncogenesis (95). HHV-8 is capable of marked modulation of the host immune system, in part due to the production of at least 10 genes with homology to human genes affecting immune function including IL-6, IL-10, TNF-alpha, CCL1, CCL2 and CCL3 (98, 99). Replication and transcription activator (RTA) protein down-regulates TLR signalling and viral interferon regulatory factors (vIRFs) block interferon signalling, reducing the innate response to the virus. vIL-6 acts *via* JAK to stimulate STAT3 and triggers a positive feedback loop generating endogenous IL-6 (99). As discussed, IL-6 is associated with MDSC generation and CCL2 with the recruitment of MDSC (100) (101).

There are relatively few studies that have looked at the immune environment in HHV-8-related tumours. In a mouse model vFLIP induced cytokine production leads to the expansion of myeloid cells with a PMN-MDSC phenotype (102). The TME in KS includes M2 macrophages, MDSCs and lymphocytes with high-level PD-L1 expression (103). In nodular KS, like in MCC, CD33+ PD-L1 positive cells have a peritumoral distribution and may function to protect the tumour from T lymphocyte infiltration (103). This study used CD33 as an MDSC marker making further phenotyping of the cells impossible and would not identify PMN-MDSC which do not act through PD-1L. Other groups have identified the PD1+ cells as being CD14+, therefore would this include monocytes and macrophages (104). In addition, HHV-8 can infect monocytes and modulate their function by upregulating PD-1 and increasing the production of a wide range of cytokines including IL-6, GM-CSF, IL-10 and IL-1beta (105). This may provide a mechanism by which HHV-8 infection of myeloid cells could trigger emergency myelopoiesis and MDSC generation.

The benefits of restoring the immune response to HHV-8 are clear; in AIDS-related KS, treating HIV with antiretroviral therapy leads to a response in 50%, although in around 10% there is a flare of disease (106). In those not responding to antiretrovirals, the standard treatment has been chemotherapy. However, there is growing evidence for immune modulation with imid drugs or PD-1 checkpoint inhibition (107). However, while HHV-8 can clearly cause conditions likely to lead to MDSC production, there is insufficient evidence of how this may impact the antiviral response or the effect of checkpoint blockade.

### Human papilloma virus (HPV)

Human papilomaviridae (HPV) are a family of small non-enveloped double-stranded DNA viruses composed of over 450 distinct types that replicate in keratinocytes of squamous epithelia of either the cutaneous or mucosal surfaces. Most HPV infections are asymptomatic or cause self-limiting benign diseases, such as warts. However, a subset of HPV types, termed ‘high risk’ are the causative agents of 1/3 of all virally induced cancers, the most common of which are anogenital cancers and oropharyngeal cancer (108). All HPV types have a similar genome divided into the early open reading frames (ORFs); E1, E2, E4, E5, E6, E7, E8, (E5 and E8 are not present in all HPV types) and the late genes L2 and L2. The core genes, E1 and E2 are required for viral replication and the L1 and L2 are required for capsid formation. The accessory genes (E4-7) facilitate the different stages of the vegetative virus life cycle, mainly by altering the host cell environment to enable viral replication but also perturbing the host anti-viral defense mechanisms (109) (108). In cervical cancer, there is a prolonged progression through increasing grades of intraepithelial neoplasia (CIN) through to invasive cancer (ICC) during which immune control is gradually lost.

The principal mechanism by which HPV evades the immune system is by taking advantage of the complex renewal process in squamous epithelial tissues. Infected basal epithelial cells initially amplify and maintain the viral episomal genome at approximately 50 – 100 copies per cell where viral DNA replication occurs synchronously with the host DNA replication. As infected cells move from the basal lamina, the viral accessory proteins act in concert to delay differentiation by promoting cell cycle re-entry and proliferation. Viral DNA replication is induced by virus-mediated de-regulation of host cell control of the cell cycle. Ultimately, keratinocyte differentiation occurs with the production of the late structural proteins, production of virus particles and the sloughing off of the virus-laden squames (110). Limiting high expression levels of the viral proteins to the uppermost differentiated keratinocytes ensures the least exposure to the host immune pathways, reduces inflammation and delays the generation of an effective immune response, although this mechanism breaks down in CIN and ICC (110). In addition, the HPV proteins exhibit some immunomodulatory functions; E5 prevents effective antigen processing and E7 downregulates class I MHC and STAT1 preventing immune signalling (111). Indeed, the majority of those infected with HPV will clear the virus, which is associated with a CD4 and CD8 response (112). However, the risk of malignancy is increased with underlying immunosuppression (113). Amongst patients with HPV16+ ICC half have no CD4+ response to E2 or E6 with the other half having an impaired response despite having a normal response to other recall antigens (114). In contrast, strong IFN-γ and IL-5 responses are seen in patients who have cleared HPV16 without developing malignancy. Prophylactic HPV vaccination leads to a strong humeral immune response against L1, which is highly effective in preventing infection and can also reduce the relapse rate following surgical removal of CIN, but is ineffective in treating advanced disease (112, 115)

There has been significant research interest in the TME in CIN and ICC, in which multiple regulatory cells including TAMs, Tregs as well as MDSCs may play a deleterious role and help explain why some patients do not mount an immune response to the virus (116). The TME in ICC is enriched with cells expressing TGF-β and IL10 with lower expression of IL2 compared to cervicitis and CIN (117). The ratio of Treg to CD4+ and CD8+ cells is higher in advanced disease with very few regulatory cells in cervicitis (117). Infiltration with CD163+ TAMs is associated with advanced-stage ICC and increases with the progression of CIN (118). Total MDSCs are expanded in the blood of patients with ICC compared to healthy controls and increase with advanced stage (119). MDSCs are also found in tumour tissue in ICC and function ex vivo to inhibit T cell proliferation and cytokine production (119). In a cohort of 22 patients with ICC MDSC numbers in both blood and tissue were associated with poorer progression free survival, although given the association with advanced stage a more sophisticated multivariate analysis with greater numbers would give additional evidence of a causal relationship (119). PMN-MDSCs appear to be the predominant population found in the blood of patients with ICC compared to those with benign cervical lesions and secrete iNOS, IDO and arginase-1 (120). Based on RNA-seq data from tumour tissue in 306 patients there was an association between high MDSC infiltration and overall survival, although again this was not a multivariate analysis (120). A further study found PMN-MDSC numbers increased with disease stage in ICC and where negatively corelated with CD8+ cells in tumour tissues (121). The role of M-MDSCs is less clear, comparing all ICC patients to those with benign lesions appears to show no increase in numbers but when ICC are sorted by disease stage M-MDSC numbers appear to increase in metastatic ICC but not early or locally advanced tumours (57, 120). In CIN there is a strong negative correlation between increased neutrophil and reduced T cell infiltration which is not seen in cervicitis (122). This indicates that the microenvironment is different in CIN compared to the inflamed cervical mucosa.

MDSC production and activation is likely to be driven by cytokine production. ICC cells secrete cytokines associated with myeloid activation and differentiation including G-CSF, IL-6, IL-10 and TGF-beta (122) (122). Plasma G-CSF concentration is associated with increased numbers of MDSCs in the blood in CIN and ICC (122). A large cohort which only looked at leucocytosis, rather than more detailed immunophenotyping, showed this was associated with a worse prognosis and correlated with immunoreactivity for G-CSF in the tumour tissue (123). G-CSF production can be detected in around 85% of tumour biopsies and is associated with a poorer response to both radiotherapy and cisplatin chemotherapy (124, 125). TGF-β signalling is increased in HPV+ tumours compared to HPV- across a range of cancer types and is associated with an inflamed TME (126). IL-10 is upregulated in high-risk cervical lesions, although upregulation in early lesions does not predict which patients progress (127). Reduced CCL2 production in ICC is associated with increased overall survival and a lower infiltrate with immunosuppressive myeloid cells, although this study identified TAMs and did not stain for MDSCs (128). HPV-E6/E7 can induce STAT-3 production in transformed keratinocytes which leads to IL-6 production. This generates a positive feedback loop by inducing STAT3 activation in monocytes which leads to further myeloid accumulation (113).

MDSCs from the blood and tumour tissues of patients with ICC can inhibit T cell cytokine secretion and proliferation (119). When neutrophils are cultured with HPV+ ICC cell lines they become activated and express IL-6, IL-8 and CD62L. When T cells are cultured with SiHa spheroids, a HPV16+ cell line, they proliferate, secrete cytokines and eliminate tumour cells. However, when neutrophils are co-cultured at a 1:1 ratio with T cells they inhibit T cell function (122). This provides strong evidence that neutrophils, activated by tumour tissue to an MDSC phenotype, can inhibit anti-tumour T cell responses. MDSCs can also act *via* other mechanisms. PGE2 can enhance the stemness of cervical cancer cells (129). MDSCs in ICC also express B cell activating factor and can induce differentiation of IL-10 producing B regulatory cells. These can act then *via* IL-10 and STAT3 to create a positive feedback loop generating further MDSCs (120)

There is substantial interest in immunotherapy for HPV malignancies including checkpoint blockade and the development of therapeutic vaccines (130). PD-1 blockade shows benefit in treating ICC and HPV positive, as well as negative, head and neck cancers, however, overall response rates are low (131, 132). Response to therapeutic vaccination is less strong in advanced disease, potentially illustrating how the TME can inhibit the immune response (130). In a murine xenograft model, an E7-based DNA vaccine can produce a CD8+ effector memory response, however, this is insufficient to eliminate the tumour. When combined with MDSC reduction using an anti-GR-1 antibody complete responses were seen (133). Combining therapeutic vaccination with all trans-retinoic acid (ATRA) is also successful in a murine model in reducing MDSC number and increasing immunogenicity (134). ATRA alone can decrease MDSC numbers and increase CD8+ T cells in BALB/C mice with U14 cervical tumours and can enhance the efficacy of PD-1 checkpoint blockade (57). In an immunocompetent murine model of cervical cancer, MDSCs accumulate. Again, therapeutic E7 vaccination can induce antigen-specific T cells but does not lead to tumour regression, an effect not enhanced with checkpoint blockade. Ex vivo MDSCs but not Tregs could inhibit T cell and antigen-presenting cell function (135). This provides strong evidence of both the effect of MDSCs in HPV-positive tumours and illustrates the difficulties they can cause with immunotherapy. Immunotherapy to eliminate suppressors may also enhance the effect of standard cancer therapy. In a murine model, using cyclophosphamide to deplete Tregs and an iNOS inhibitor to reduce MDSC function alongside radiotherapy had a beneficial effect. This included a greater response to treatment as well as a significant increase in antigen specific CD8+ cells in the tumour (136).

Overall, the evidence for the role of MDSCs in HPV tumours is strong. In early-stage HPV infection virus can hide from the immune system due to its location combined with suppression of TLR and MHC expression. As CIN develops a cytokine milieu leads to the generation and invasion of regulatory cells including MDSCs. Most patients can clear the virus, presumably because the cytotoxic response can overpower immunosuppressive. However, in those with cancers, the immunosuppressive response predominates. There is still the need however for further data. There is no human data on the role of the MDSCs in predicting response to immunotherapy in HPV+ cancers, unlike what is seen in murine models. There is also insufficient data showing a clear correlation between MDSC expansion and disease progression, rather than an association with advanced disease.

### Hepatitis B virus (HBV)

Hepatitis B virus (HBV) is a member of *Hepadnaviridae* with a partially double-stranded DNA genome which is transmitted by infected bodily fluids and replicates *via* reverse transcription (137). In low-prevalence countries infection is usually sexually transmitted in adulthood, triggering a strong immune response. This leads to acute hepatitis followed by viral clearance in 99% of patients. In high-prevalence areas infection is perinatal, this leads to a tolerogenic immune response with viral persistence in 90% (138). Immune response to HBV in chronic infection is complex (139). Initially, the immune response is tolerogenic (IT) with high viral loads and low levels of CD8 T cell activation. Over decades the CD8 response becomes stronger leading to chronic hepatitis (CHB) with intermittent flairs of acute hepatitis (ACLF) and liver damage. Some patients clear most infected hepatocytes and seroconvert to produce anti-HBV E antigen (HBeAg) with low levels of HBV DNA and slowing of the progression of liver injury. In around 1% of anti-HBeAg positive patients per year, the viral DNA becomes undetectable, indicating a functional cure. The rest develop cirrhosis or hepatocellular carcinoma (HCC) many years after the initial infection (140).

HBV can directly lead to oncogenesis through random integrations into the human genome after reverse transcription, this increases genetic instability and prolongs viral gene expression (140). The HBV X antigen (HBx) can interact with transcription factors such as CREB promoting transformation (141, 142) and epigenetically silence tumour suppressors such as RUNX3, p16 and p21 through promoter hypermethylation (143, 144). Indirectly, HBV-associated chronic inflammation leads to oxidative stress, ROS production and altered cellular metabolism promoting DNA damage and leading to oncogenesis (145, 146).

The immune response to HBV includes innate immune IFN production, NK and T cells acting in both cytolytic and non-cytolytic manners against infected hepatocytes as well as the production of neutralizing antibodies by plasma cells (147). The tolerogenic state seen in chronic infection is characterized by regulatory and exhausted cells being enriched in the liver microenvironment including FOXP3^+^ T cells, PD-1/PD-L1 axis activation and the production of IL-10 and TGF-β with a weak CTL response to HBV antigens (148). However, the mechanisms behind this dysfunctional response are not fully understood. MDSCs are significantly expanded in CHB patients (60, 149–154), indicating a potential role in preventing viral clearance.

The relative importance of PMN- and M-MDSCs in CHB is debated, with divergent findings in different studies. Both the analytical strategies and dynamics during the progression of CHB might contribute to these discrepancies. M-MDSCs have been reported as being expanded in Indian and Chinese cohorts (155) (154, 156), whereas in a European cohort PMN-MDSCs were expanded more significantly (60). In paediatric, but not adult, CHB patients M-MDSC numbers correlated inversely with CD8 responses to HBsAg (157). These cells could home to the thymus and when transferred to mice could selectively inhibit HBsAg targeting CTLs. In ACLF M-MDSC expansion appears to correlate with markers of liver damage but not viral replication (153). Whereas in chronic infection DNA load and HBeAg positivity appears to be more significant (151, 154, 156). The relationship between MDSCs and liver damage is not clear with some groups showing a positive correlation with increasing hepatitis and others a negative implying a protective role (151, 154). MDSC phenotypes and function appear to dynamically change over time in different stages of HBV infection (60). Profound suppressive activities, including expression of Arginase, iNOS and PD-L1 are seen in active CHB, but much weaker suppressive functions, expressing only arginase, in the IT state (152). PMN-MDSCs expand transiently in acute HBV and are increased most in disease states with high viral replication but low levels of immune-mediated inflammation (60). This emphasizes that PMN-MDSCs may prevent immunopathology in CHB, but at the expense of poor viral control.

There is much less data on the role of MDSCs following the development of HCC. Elevated LOX-1^+^CD15^+^ PMN-MDSCs in both circulation and tumour tissues were correlated with poor clinical outcomes in HCC, but not with HBsAg expression (158). Expansion of M-MDSCs in HCC has also been associated with a poor prognosis after surgical or chemotherapeutic treatments (159–161). Only a single study has described increasing numbers of both PMN-MDSCs and M-MDSC on the progression of CHB to HCC with a positive correlation with increased liver damage (149). Interestingly, this study did not find any association of MDSC numbers with other clinical features or OS, which may be a function of relatively low patient numbers. More data are needed, with prospective follow-up, to examine if MDSC numbers in CHB are a risk factor for future HCC development.

MDSCs have been reported to act *via* several mechanisms in CHB infection. M-MDSCs can act *via* PD-1/PD-L1 and IL-10 to suppress HBsAg-specific cytotoxic function and proliferation in CD8+ T cells (154). PMN-MDSC from CHB patients inhibits T cell function in an arginase-dependent manner *in-vitro*. Arginase expressing MDSC can be identified in the blood and liver of CHB patients (60). *In-vitro* treatment of PBMC with HBeAg induces IDO expressing M-MDSCs which inhibit CD4^+^ and CD8^+^ T cell proliferation and IFN-γ production (156) Tregs can be induced by MDSCs in HBV infection in an IL-10 and TGF-β dependent process (155). In a mouse model, chemokine receptor CCR9 in M-MDSCs is induced by HBsAg *via* ERK/IL-6 pathway, and CCR9-CCL25 interaction mediates Gr1^+^Ly6C^high^Ly6G^-^ M-MDSCs homing to the thymus, where HBsAg-specific CD8^+^CD4^-^ T cells are killed by NOX1-expressing M-MDSCs (162). Another HBV-specific mechanism involves impaired CCR5-CCL5 interaction by TGF-β released from MDSCs, which affects the migration of HBV-specific T cells to the liver (152, 163, 164). This effect is seen during active hepatitis, but not during IT or after viral clearance (152).

Hepatitis antigens appear to be capable of directly inducing MDSC production. When PBMC are treated with HBeAg *in-vitro* there is induction of IL-1β, IL6 and IL-10 along with the accumulation of IDO producing MDSCs. Blocking IL-6 or IL-1β with neutralizing antibodies prevents MDSC generation (156). HBsAg can induce arginase and NOX-1 producing MDSC differentiation from mature monocytes *via* an ERK/IL-6/STAT-3 dependent process (165). It is interesting that different viral proteins can produce MDSC with altered function and illustrates the potential complexity of the immune microenvironment shaped by HBV infection. HBx and HBcAG can induce hepatocytes to secrete IL-6 which is likely to be capable of inducing MDSCs in a paracrine manner (166, 167). In a murine model of CHB, γδT cells can induce CD11b^+^Gr1^+^ M-MDSCs in mice through an IL-17-dependent manner, which then inhibit IFN-γ production by hepatic CD8^+^ T cells *via* ARG-I expression (168).

Therapy for HBV consists of either nucleoside analogue antiviral drugs such as entecavir, achieving viral clearance in 67% (169), or peg-IFN-α-a2 with a clearance rate of 25% (170). Even after viral clearance, Tenofovir does not normalize MDSC or Tregs numbers and HBV-specific CTL responses remain impaired (152, 155). This indicates that viral clearance is not necessarily correlated with recovered immune responses and may allow viral rebound at the end of treatment. This creates a rationale for combing immunotherapy with direct antivirals (171). Knocking down HBx expression with a siRNA can reduce MDSC frequency, IL-10 and ROS level in HBV-infected mice (172). Activating TLR-8 with GS-9688, a specific agonist decreases MDSC and Treg numbers and promotes a cellular antiviral response using ex vivo cells from CHB patients. This can reduce viral loads in a woodchuck HBV model (173). However, the residual MDSCs released more immunosuppressive gelactin-9 and PD-L1 after treatment (173). While IFN-α has been widely used in CHB treatment, its immunological effects are complex and may promote MDSC production (174). IFN-α can increase Tregs and MDSCs numbers with increased IL-10 and PD-1L expression, while the roles of Tregs and MDSCs promote tolerance in acute hepatitis B in a murine model (175). ATRA can reduce HBsAg-induced M-MDSC function and restore T cell function in a murine CHB model (165). Icaritin, a small molecule derived from horny goat weed, can prohibit the generation of MDSCs by disrupting STAT3 and AKT signalling. This can then modulate cytokine secretion in HCC patients (176). Again, in a murine model, depleting MDSCs with an anti-GR1 antibody or inhibiting their function with an arginase inhibitor can enhance CTL function and promote viral clearance (168).

Overall, there is convincing evidence for a functional role of MDSCs in HBV infection but the significance of this cell population in preventing viral clearance or allowing the development of HCC is unclear. Investigating MDSCs in HBV is difficult given the different stages of infection, where these cells may have different functional roles. Prospective trials where the same patients have measurements of MDSC phenotype and number in different phases of their disease using a standardized methodology may help answer these questions. This may also help identify if targeting MDSCs would be an effective method to help with viral clearance and prevent the development of HCC.

### Hepatitis C virus (HCV)

The Hepatitis C virus (HCV) belongs to *Flaviviridae* and has a single-stranded RNA genome (177). HCV is transmitted by infected blood exposure, which can be through transfusion, IV drug use or vertical transmission, and leads to chronic hepatitis (CHC) in 70-80% of those infected (178). 10-20% of those with CHC develop cirrhosis over 20-30 years and HCV is the second most common cause of HCC after HBV (179). There is no effective vaccine to prevent HCV infection, but treatment has been revolutionised over the last 10 years with direct-acting antivirals (DAA), which can cure 90% of patients (180, 181). Unlike HBV, HCV-associated HCC always develops occurs in the context of cirrhosis and HCV does not appear capable of direct transformation of hepatocytes (140). Instead, cytotoxicity mediated by type I IFN, NK cells and CD8^+^ effector T cells generates an inflammation-necrosis-regeneration cycle with ROS production, triggering lipid peroxidation, DNA damage and mitochondrial dysfunction (182, 183). Additionally, the HCV core antigen (HCVcAg) activates the Ras/MAPK and PI3K/Akt pathways which can promote transformation (184, 185). HCVcAg also plays a role in metabolic reprogramming with enhanced lipogenesis and impaired lipid degradation, this promotes steatosis and the development of cirrhosis (186).

During acute HCV infection, there is a robust IFN response triggered by HCV RNA and antigens through RIG-I and TLRs (187, 188). Viral clearance depends on a strong cellular and humoral adaptive immune response followed by the development of memory (189). However, in most patients a combination of immune exhaustion, regulatory cell expansion and viral escape mutations causes this process to fail (189).

MDSC may act as part of the expanded regulatory population in CHC (190–201). M-MDSC are the population most frequently reported as being elevated (190–195), with a few groups also observing PMN-MDSC expansion (196–200). Most studies have not attempted to study PMN-MDSCs, and others have carried out flow cytometry on frozen cells, which adversely affects PMN-MDSC enumeration as discussed earlier (190). It is unclear how much this variation is explained by biological variation in heterogenous patient populations or is a result of methodological differences

The clinical significance of MDSCs in CHC has also been explored. Many research groups identified positive correlations between MDSC frequencies and HCV genotypes (196), HCV RNA load (191, 192, 195, 196, 200, 201) or liver damage parameters including ALT and AST level (191, 195, 201), and negative correlation with CD4^+^ or CD8^+^ T cell counts and functions (191, 201). Although not all studies have found these associations (194, 197). It may be that MDSC populations change dynamically in response to viral factors, inflammation, development of cirrhosis and treatment as described in HBV (152). Longitudinal analysis of MDSC populations in different phases of disease and treatment may help better describe the significance of this population The role of HCV in producing MDSCs in HCC is another key question. One study compared PMN-MDSC numbers in HCC patients with and without HCV infection in an American patient cohort (202). MDSCs and Tregs were increased in HCC patients compared to healthy volunteers but there was no difference between virus positive and negative cases. This may relate to the presence of multiple factors capable of generating MDSCs being present in established HCC. It would be interesting to see similar work in other populations as well as to assess if this is also true for M-MDSCs.

PMBCs which have been exposed to HCV can develop an M-MDSC phenotype and reduce NK IFN production through arginase depletion which then suppresses mTOR signalling (203). Exposing CD33+ cells to HCVcAg or infected hepatocytes generates M-MDSCs, which upregulate ROS but not ARG-1 or iNOS through a p47-dependent process and then inhibit CD4 and CD8 T cells (204). HCV-induced M-MDSCs express high levels of pSTAT3 and Il-10 and can induce Treg function (194) MDSCs isolated from CHC patients can increase the ratio of follicular regulatory to follicular helper T cells in a cell contact-dependent process (205). This may then inhibit T cell-dependent B cell function.

PMN-MDSCs can be generated from PBMCs by culturing with HCVcAg in a process reliant on STAT3 which is activated by ERK 1/2 (201), However, M-MDSC generation is more reliant on the TLR2/PI3K/AKT/STAT3 axis, resulting in upregulated IDO, PD-L1 and IL-10 (206, 207). This suggests that different mechanisms are involved in M-MDSCs and PMN-MDSCs generation, although both are induced by HCVcAg. Interestingly, exosomes containing viral RNA generated by HCV-infected hepatocytes can also induce the generation of M-MDSCs (198, 199, 205). This process relies on modulating HOTAIRM1, HOXA and miR124 and leads to an M-MDSC phenotype with STAT3 activation and production of iNOS and ARG-1. An attempt to develop an HCV vaccine using mice mesenchymal stem cells transfected to express non-structural HCV proteins (NS3~NS5B) triggers strong anti-HCV immunity without expanding MDSC (208). Suggesting that this may be a successful approach for vaccine development.

During treatment with IFN and ribavirin, MDSC numbers decrease in proportion to HCV RNA load. This occurs as early as 4 weeks into therapy and is accompanied by increases in T cell function (191, 196, 197). After IFN therapy M-MDSC numbers rise with higher levels seen in non-responders versus responders (209). Although, the significance of this is less relevant as this therapy has been superseded by direct activating antivirals. Results with DAA have been mixed. A study looking at a European population did not detect significantly reduced M-MDSCs until 48 weeks post-therapy, along with scant recovery of T cell functions (193). However, in a Chinese cohort, there was a rapid reduction in M-MDSCs along with restoration of CD8^+^ T cells and NK cells with DAA therapy at as early as 12 weeks. The authors proposed the recovery of immune cells was associated with efficient viral clearance achieved by DAA therapy (195). In another Chinese cohort, PMN MDSCs but not M-MDSCs were elevated after DAA with levels higher in those not achieving a sustained antiviral response (201). These studies used different flow strategies, were carried out in different populations, may have involved infections with different strains of HCV and there was no detailed discussion in either paper of the degree of underlying liver damage in their respective cohorts. It may be that immune recovery in HCV requires both viral clearance and resolution of inflammation.

Overall, our current understanding of MDSCs in HCV infection, including their presence, clinical significance, immunosuppressive properties and generation are limited. Although there is data demonstrating the association between MDSC expansion and disease parameters, some results are conflicting. To build on this it will be necessary to use standardized MDSC detection techniques and correlate with clinical and virological features. There is also a need for further data on the link between MDSCs and HCC in CHC. This may better help understand the propensity of HCV to cause chronic infection. Furthermore, understanding the ability of HCV to cause sustained immune changes may help guide how antiviral and immunotherapy can be combined.

### Epstein Barr virus (EBV)

EBV is a γ-1 herpesvirus which infects 90-95% of individuals worldwide, establishing a lifelong latent infection. Primary infection is classically responsible for infectious mononucleosis, although infection in childhood is usually asymptomatic. EBV has a natural tropism for B cells but can also infect T cells and epithelial cells and is causally linked to a range of cancers including lymphoma, nasopharyngeal cancer (NPC) and gastric cancer (210).

After primary B cell infection, EBV initiates the expression of a unique set of growth transforming genes including the virus-encoded nuclear antigens EBNA 1, 2, 3A, 3B, 3C, and –LP, the latent membrane proteins LMPs 1 and 2A/2B, the non-coding EBER RNAs and two blocks of microRNAs (BHRF1- and BART-miRs). This growth transforming Latency III is highly immunogenic and vulnerable to immunosurveillance, therefore to establish lifelong persistence, the virus adopts a restricted Latency 0 expressing only the EBERs and BART-miRs (210). However, this reflects only two of several alternative forms of latency with increasingly restricted transcriptional programmes compared to Latency III. These alternate forms of latency, termed Latency II and I are observed during the establishment of Latency 0, however all forms of latency have been observed in the EBV-associated malignancies and are dependent upon the cell type infected and the class of tumour. Most EBV-related Hodgkin (HL) and non-Hodgkin lymphomas (NHL) as well as epithelial malignancies are latency II, (EBNA-1, LMP-1-2, EBER, BART-miRs), which rely on both EBV genes and somatic mutations to drive tumour growth (211). Importantly latency III tumours generally only occur in immunosuppressed states such as post-transplant lymphoproliferative disease (PTLD).

In healthy carriers, CD8+ responses to EBNA-3s are the dominant, with CD8+ responses to EBNA-1, EBNA-2 and LMP-2 subdominant and rare responses to LMP-1 (212). While latency I and II tumours are more common in immunosuppression, they also occur in those with intact immune systems where they virus employs a plethora of immune modulatory mechanisms. Such mechanisms include EBER-mediated induction of IL-10 in B cells (213); exosomes produced by NPC inducing Treg migration to the TME *via* CCL20 (214); inhibition of interferon signalling and reduction of class II MHC expression by BZLF1 and BRLF1 (215, 216), amongst many others. In addition, there is increasing evidence of a role for MDSC in driving immune evasion.

Both PMN-MDSCs and M-MDSCs have been identified in patients with EBV-related tumours. MDSCs, predominantly of a monocytic phenotype, are expanded in the blood of ENKTL patients (213). Ex vivo these cells can suppress T cell proliferation and IFN-γ production. In a multivariate analysis, high levels of MDSCs are associated with a worse prognosis in ENKTL (213). In chronic active EBV (CAEBV) PMN-MDSCs are expanded and again functionally inhibit T cell responses (217). Interestingly, while LMP-1-specific CTLs were isolated from these patients, no anti-tumour immune response was seen. MDSCs are expanded in NPC cancer patients and correlate with disease burden. In one study enrolling 49 patients’ total blood and tumour MDSC levels were associated with a worse prognosis (218). Unfortunately, this study did not use standardised flow to identify MDSC subtypes and did not carry out a multivariate analysis

There are multiple potential drivers of MDSC production and differentiation. High levels of the MDSC-associated cytokines GM-CSF, IL-1beta and IL-6 are seen in CAEBV patients with the same cytokines produced by CAEBV cell lines (217). In NPC, MDSC levels correlated with COX-2 production by the tumour and knocking down COX-2 could inhibit MDSC production *in-vitro* (218). High levels of the MDSC-associated cytokines GM-CSF, IL1β and IL-6 are seen in CAEBV patients with the same cytokines produced by CAEBV cell lines (217). In NPC, MDSC levels correlated with COX-2 production by the tumour and knocking down COX-2 could inhibit MDSC production *in-vitro* (218). In a murine model, MDSCs could stimulate further COX-2 expression by the NPC cells, an effect which could be blocked by inhibiting TGF-β or iNOS and enhanced through arginine supplementation. LMP-1 appears capable of inducing extra-mitochondrial glycolysis through a GLUT1-dependent process. In NPC tissues there is a strong correlation between LMP1 and GLUT1 with numbers of CD33+ MDSC. GLUT1 glycolysis activates the phosphorylation of NF-kB, induces COX-2 signalling pathways and activates the NLRp3 inflammasome leading to the production of IL-1β, IL-6 and GM-CSF (219). EBV may be able to directly modulate myeloid cells. EBV can infect monocyte-derived macrophages *in-vitro* and increases the production of IL-6 and TNF-α which then induces IDO (220). However, it should be noted that PMN-MDSCs and M-MDSCs are expanded in HL and NHL regardless of aetiology and are associated with a worse prognosis (221, 222). Therefore, the mechanism for MDSC generation cannot purely be a direct effect of the virus. Unfortunately, there have been no comparisons between EBV-positive and negative lymphoma or NPC which have looked at differences in MDSC populations or drivers for their differentiation.

There is the potential for crosstalk between cancer-causing viral infections which can drive MDSC generation. More NPC patients with CHB have EBV-positive tumours than those without this condition. CHB is associated with increased LOX1+ MDSCs and higher levels of EBV DNA (219). This implies that hepatitis B-induced MDSCs may inhibit the immune response to EBV, although further evidence would be needed to demonstrate this conclusively.

There is substantial interest in using EBV-targeted CTLs to treat EBV-positive tumours (223). However, response rates in advanced tumours other than PTLD may be limited (224). A clinical trial in NPC gave chemotherapy followed by multiple infusions of CTLs. EBV viral load and a high monocyte-to-lymphocyte ratio were associated with poor survival (225). The patients with the best responses showed a CD8+ cytotoxic response which was not seen in poor responders who had an expansion of M-MDSCs. Unfortunately, flow cytometry was performed retrospectively on frozen samples precluding analysis of PMN-MDSCs. Unfortunately, no other studies have examined MDSCs in the context of CTL therapy so the evidence for this is limited.

Overall, there is good evidence that MDSCs play a role in inhibiting the immune response to EBV in NPC and EBV-related T-cell lymphomas. The data for the role of EBV in modulating MDSCs in the TME of other lymphoma subtypes is much more limited. Answering these questions will require comparisons between EBV-positive and negative tumours. This may help guide the use of EBV-targeted immunotherapies.

## MDSC targeted therapy

Given their association with poor prognosis in multiple cancer types, there have been efforts to target MDSCs to improve the outcomes of cancer therapy. Potential strategies include depletion of MDSC populations, inhibiting recruitment to the TME, blocking suppressive functions, and differentiating away from a suppressive phenotype (226). The potential approaches, along with the evidence for them, are summarised in **Table 2**. However, very little of this research has been focused in the context of virally induced cancers or how therapy may impact the transformation of premalignant lesions.

**Table 2:** Approaches to modulate MDSC populations in cancer.

Strategy	Rational	Evidence	Ongoing clinical trials
Gemcitabine	Chemotherapeutic agent believed to have a specific MDSC depleting effect	Mixed evidence- reductions in MDSC numbers seen in some tumour types and not others (227)	NCT02479230- Gemcitabine with dendritic cell vaccine in breast cancer Drug in clinical use in multiple cancer types as chemotherapeutic agent
Gemtuzumab Ozogamicin	Antibody drug conjugate targeting CD33	Effective *in vitro* (228)	No specific trials investigating MDSCs Drug in clinical use in Acute Myeloid Leukaemia
COX-2 inhibition	Reduces PGE2 production and prevents effect on myeloid cells	Effective in animal models in combination with immunotherapy (229) Improves outcomes in human cancers through multiple potential mechanisms (230)	NCT03245489- Assessing COX-2 inhibition along with other anti-platelet therapy in combination with checkpoint blockade in head and neck cancer
ATRA	Induces MDSC differentiation away from suppressive phenotype	Improves responses to checkpoint blockade in a murine model of cervical cancer (57) Can reduce MDSC populations in combination with checkpoint blockade in melanoma. No evidence of improved efficacy of treatment (231).	NCT04919369-ATRA and checkpoint blockade in lung cancer NCT05388487- Pegylated ATRA in multiple refractory cancers
PDE-5 inhibitors	Downregulates iNOS	Modulates the TME and reduces MDSCs in head and neck cancers (232). No evidence of improved outcomes in patients. Trial terminated for futility in myeloma (233)	NCT02544880- PDE5 inhibition combined with tumour vaccine NCT05709574- PDE5 inhibition combined with chemotherapy in gastric cancer
CCL2 blockade	Prevents MDSC migration to tissues	Carlumab, a CCL2 targeting antibody is ineffective as a single agent in cancer (234)	No currently recruiting trials
STAT3	STAT3 signalling is important in MDSC suppressive function	Effective in murine melanoma model in reducing immunosuppressive effect of MDSCs (235) Not effective in cancer outcomes in currently reported trials which did not assess for MDSC effects (236)	No currently recruiting trials
PI3K	PI3K-γ is highly expressed in tumour infiltrating myeloid cells and promotes migration and immunosuppressive function	Inhibits MDSC function *in vitro* and synergises with checkpoint blockade in murine models (237)	NCT03673787- Ipatasertib in combination with checkpoint blockade in advanced solid tumours NCT03961698- Eganelisib with checkpoint blockade and chemotherapy in renal and breast cancer

Some chemotherapeutic agents may be able to reduce MDSC populations. Gemcitabine (GEM) appears capable of reducing PMN-MDSCs in patients with pancreatic cancer, although the effect is transient and M-MDSCs are not affected (238). Although, the evidence is mixed as GEM appears capable of enhancing the function of M-MDSCs in a mouse breast cancer model (239). The CD33 targeting antibody-drug conjugate gemtuzumab ozogamicin can efficiently eliminate MDSCs, however, it may be too toxic for easy combination with other treatments (228, 240)

In multiple cancer models including EBV-related NPC, COX appears to be a marker for immune evasion partially through PGE2-driven effects on myeloid cells (218, 241). COX-2 inhibition can decrease MDSC numbers and increase the efficacy of dendritic cell-based immunotherapy in the context of a murine model of mesothelioma (229). PGE2 induces arginase expression in a mouse model of lung cancer and inhibiting COX-2 can lead to a lymphocyte-mediated anti-tumour response (242). A large meta-analysis, across multiple tumour types, showed modest benefits (238) in adding the selective COX-2 inhibitor celecoxib to standard anticancer therapy with no concerning toxicity (230). Celecoxib has been used in a phase I trial combined with radiotherapy in NPC with encouraging outcomes and no clear toxicity, however, this trial did not report on the effect of EBV positive versus negative tumours or look at MDSC numbers or function (243). Overall, COX-2 inhibition is an interesting strategy given its low toxicity and data on combination with immunotherapy in virally induced cancers would be interesting.

As discussed earlier, ATRA can decrease the frequency of MDSCs, improving response to checkpoint blockade or therapeutic vaccination in murine models of cervical cancer (57, 134). One small clinical trial showed that ATRA can decrease the frequency of MDSCs and appears to increase T cell responses to checkpoint inhibition, although this was in the context of melanoma. Unfortunately, this study was too small to see if this led to improved tumour responses (231). ATRA acts by inducing MDSC differentiation away from a suppressive phenotype through the upregulation of glutathione synthesis (244). Other potential therapeutic approaches include phosphodiesterase-5 (PDE-5) inhibitors, which can reduce MDSC numbers in head and neck cancers through the downregulation of iNOS (232). Blocking chemokine signalling has the potential to prevent MDSC accumulation in the tissues. However, the CCL2 targeting MAB carlumab was unable to affect the CCL2/CCR2 axis sufficiently or have a single agent anti-tumour effect (234). Inhibiting STAT3 is another potential method to reduce the effect of MDSCs. The STAT3 inhibitor Napabucasin could eliminate the immunosuppressive effects of MDSCs in a murine model and *in-vitro* with human M-MDSCs (235). Although, this will require the use of highly selective inhibitors to avoid inhibiting STAT-dependent T cell function (245). PI3K, especially the PI3K-γ isoform is highly expressed on tumour infiltrating myeloid cells. Pharmacological inhibition of PI3K-γ can restore sensitivity to checkpoint blockade in murine cancer models (237). Although when targeting PI3K it is important to use specific inhibitors at appropriate doses to prevent off target inhibition of T cell function (246).

Targeting MDSCs appears to be an attractive therapeutic strategy. However, while there are options available, all are at a relatively early stage in development. The true potential in targeting MDSCs may come from a combination with other immunotherapy options.

## Discussion

There is growing evidence that MDSC expansion could help to explain how cancer-causing viruses can evade immune detection, trigger oncogenesis and prevent immune recognition of the resultant tumour, although the strength of evidence for this is highly dependent on the virus type. As illustrated in **Figure 1**, many factors released from virally transformed or infected cells along with substances directly produced by cancer-causing viruses are linked to the pathways known to generate MDSCs. There are, however, several unanswered questions. How much of the expansion in MDSCs is a direct result of the viral infection, which may occur at an early stage in infection, or a result of cellular transformation? For this question, comparisons of the TME between viral and non-viral mediated types of the same tumour may be beneficial. Is MDSC expansion a marker of advanced malignancy or a true independent marker of poor prognosis? How significant is the impact of MDSCs on targeted immunotherapy? This last question is becoming more significant given the development of therapeutic vaccines or cellular immunotherapies.

MDSCs remain challenging to study. In the blood human PMN-MDSCs can only be reliably identified with density gradient centrifugation followed by multicolour flow which has to be performed on fresh specimens. In tissues, MDSC identification requires the use of multiple different markers. There is also a need to correlate MDSC numbers and function with responses to treatments, particularly with novel immunotherapies. This will allow the potential benefits of MDSC manipulating treatments to be ascertained. However, doing this effectively will require the examination of larger cohorts of patients alongside other clinical data. However, there is scope for progress as newer highly multiplexed techniques may make the identification of MDSCs alongside the study of the tumour and other cells in the TME easier. Hopefully, this approach will help conclusively determine the role of MDSCs in virally induced cancers.

## Author contributions

CSL and AG conceived the paper. All authors listed made a substantial, direct and intellectual contribution to the work and approved it for publication.
